# A Bibliometric Analysis of the Highest Cited Rhytidectomy Literature

**DOI:** 10.1093/asjof/ojad099

**Published:** 2023-10-31

**Authors:** Atul Dutt, Ajay P Dutt, Rohin K Reddy, Walton N Charles, Hamid Reza Khademi Mansour, Foad Nahai, Ankur Khajuria

## Abstract

In this bibliometric analysis, we investigated the top 100 most cited articles on rhytidectomy, a prevalent cosmetic surgical procedure in the United States of America. Using data from Web of Science spanning from 1900 to 2021, we found these papers collectively cited 7737 times, with individual citation counts ranging from 277 to 37 (mean 77). Notably, the majority of these papers (58 out of 100) were categorized as Level of Evidence 5, indicating a prevalence of expert opinions, anatomical studies, and narrative reviews. Interestingly, none of the papers achieved Level 1 status, underscoring a lack of high-quality research in the field. The primary focus of these papers was on operative techniques (48 papers) and surgical anatomy of the face (20 papers). Only 10 articles incorporated patient-reported outcome measures (PROMs), but none utilized validated scales. This analysis highlights the urgent need for improved research methodologies in rhytidectomy studies, emphasizing the necessity for rigorous, high-quality research, and the implementation of validated rhytidectomy-specific PROMs.

**Level of Evidence: 3:**

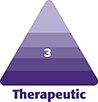

Age-related changes in facial appearance are inevitable, with the pursuit of a more youthful facial appearance forming one of the primary reasons why many undergo cosmetic procedures. Rhytidectomy, or facelift surgery, is the mainstay of facial rejuvenation and is among the top 5 cosmetic surgical procedures performed in the United States of America.^1^ The goal is to reverse the aging changes through the mobilization and fixation of deep tissues and skin.^2^ In the hundred or so years since its inception, the procedure has undergone incremental evolution, driven by a combination of increased knowledge of the intricate anatomy, a clearer understanding of the aging process, and the rise of minimally invasive techniques.^3^

Bibliometric analysis provides perspective on the growth and impact of scientific literature, when combined with citation analysis, outlines the productivity, and impact of the published material.^4^ Thus, bibliometric analysis facilitates both qualitative and quantitative evaluation of published literature, providing unique insights into influential publications within a given field.

A previous bibliometric analysis of the rhytidectomy literature through K-means clustering, a machine-learning approach, defined 2 keyword clusters: “surgical outcomes and techniques/approaches” and “study design.”^3^ However, no further work was done to characterize the contents of these broad thematic clusters. Additionally, previous bibliometric analyses of the general plastic surgery literature have revealed low use of validated objective cosmetic outcome measures^5^ and low levels of evidence.^5-8^ The quality of the most cited rhytidectomy literature remains uncertain, so we hypothesized that similar trends may exist. Therefore, our goal in undertaking this bibliometric analysis of the peer-reviewed literature was aimed at building upon the work of Honeybrook et al^3^ by discerning and defining the trends, characteristics, and quality of the top 100 most cited articles.

## METHODS

A literature search of all journals and years (1900-2021) available on the online database Web of Science (version 5.35; Clarivate Analytics, Philadelphia, PA) was performed on November 25, 2021. Searching the terms “facelift” and “rhytidectomy,” the “topic” search yielded 1891 articles.

**Figure 1.**
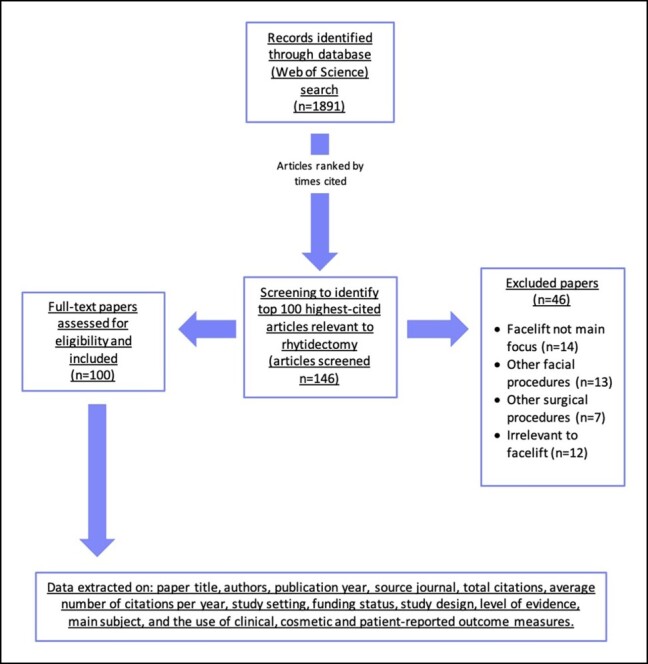
Flow chart summarizing methodology.

The 1891 papers were subsequently sorted by “times cited” in descending order. Articles with an identical number of citations were distinguished by the mean number of citations per year, with more recent papers achieving a higher ranking. Two independent reviewers (coauthors A.D. and A.P.D.) screened the titles and abstracts of papers to compile the list of the 100 most highly cited papers directly relevant to rhytidectomy. Discrepancies were resolved by discussion with the senior author (A.K.), with review of full-text publications to settle any remaining doubts. One hundred and forty-six papers were screened to compile the list of 100 most highly cited articles on rhytidectomy for inclusion. Figure 1 specifies the reasons why screened articles were excluded.

Data extraction from full-text articles was independently undertaken by 2 authors (A.d. and A.P.D.), and included: article title, authors, year of publication, journal source, total citations, mean citations per year, study design, study setting, funding status, level of evidence (graded as per the Oxford Centre for Evidence-Based Medicine [OCEBM] system [2011]),^9^ main research theme, and the use of clinical, cosmetic, and patient-reported outcome measures (PROMs). The extracted data were entered into a standardized computer spreadsheet (Excel version 16.0; Microsoft Corporation, Redmond, WA).

## RESULTS

### Citation Analysis

The 100 most highly cited articles on rhytidectomy were cited by 7737 articles (see the Appendix, which displays the references to the 100 most cited articles). The number of citations accrued per paper ranged from 277 to 37. Articles were cited on average 77 times, with the mean number of citations per article per year ranging from 9.8 to 0.7 (Table 1).

**Table 1.** The 100 Most Cited Papers in Rhytidectomy

Rank	Study	Total citations	Mean citations/year
1	Stuzin et al^1^	277	9.55
2	Hamra^2^	264	9.10
3	Hamra^3^	254	8.19
4	Hamra^4^	237	9.12
5	Mendelson et al^5^	176	9.26
6	Baker and Conley^6^	168	4.00
7	Alsarraf^7^	167	7.95
8	Owsley^8^	152	5.43
9	Moss et al^9^	135	6.43
10	Hester et al10	133	6.33
11	Hamra11	132	5.74
12	Tonnard et al12	125	6.58
13	Kosowski et al13	118	9.83
14	Hamra14	116	4.64
15	Stuzin et al15	110	4.23
16	Gosain et al16	109	6.81
17	Grover, et al17	106	5.30
18	Ramirez18	106	3.93
19	Chang et al19	104	4.16
20	Connell20	102	2.37
21	Zhang et al21	102	5.37
22	Riefkohl et al22	99	2.83
23	Marchac and Sandor23	96	3.56
24	Kikkawa et al24	95	3.80
25	Barton25	88	3.03
26	Ghassemi et al26	86	4.78
27	Hamra27	86	2.97
28	Ruiz-Esparza and Gomez28	85	4.72
29	Ivy et al29	83	3.32
30	Barton30	83	2.86
31	Little31	83	3.95
32	Owsley32	81	1.84
33	Jones and Grover33	80	4.71
34	Gosain et al34	80	3.20
35	Tellioglu et al35	80	3.81
36	Mendelson et al36	77	5.92
37	Hamra37	76	4.47
38	Ramirez38	74	3.89
39	Mendelson39	73	3.65
40	Owsley40	71	1.87
41	Baker et al41	71	4.44
42	Lemmon and Hamra42	69	1.68
43	Mckinney and Katrana43	68	1.66
44	Stuzin44	67	4.79
45	Wong and Mendelson45	67	8.38
46	Labbe et al46	65	4.33
47	Byrd and Andochick47	63	2.52
48	Rohrich and Beran48	63	2.63
49	Daane and Owsley49	61	3.39
50	Baker et al50	60	1.36
51	Hamra51	59	2.57
52	Trussler et al^52^	58	5.27
53	Rees^53^	58	2.15
54	Baker^54^	58	1.53
55	Marten^55^	55	4.23
56	Lambros^56^	55	4.23
57	Ozdemir et al^57^	55	2.89
58	Seeley et al^58^	55	3.67
59	Rohrich et al^59^	55	4.58
60	Goin et al^60^	52	1.27
61	Delaplaza et al^61^	51	1.70
62	Gassner et al^62^	49	3.77
63	Kamer and Frankel^63^	49	2.13
64	Paul et al^64^	49	3.27
65	Harshai et al^65^	48	1.92
66	Mendelson^66^	48	1.85
67	Jones and Grover^67^	47	2.76
68	Hamra^68^	47	2.47
69	Sasaki and Cohen^69^	47	2.47
70	Owsley and Fiala^70^	47	1.96
71	Guyuron and Vaughan^71^	47	1.62
72	Rees et al^72^	47	0.98
73	Berner et al^73^	46	1.02
74	Fezza et al^74^	46	2.42
75	Ramirez^75^	45	1.55
76	Warren et al^76^	45	4.50
77	Jones et al^77^	45	3.21
78	Hamra^78^	44	1.42
79	Stuzin et al^79^	44	2.10
80	Freiberg et al^80^	44	1.83
81	Pitanguy^81^	44	1.10
82	Gunter and Hackney^82^	43	1.95
83	Teimourian^83^	43	1.13
84	Barton and Gyimesi^84^	43	1.79
85	Matarasso and Terino^85^	43	1.59
86	Conway^86^	42	0.82
87	Macchi et al^87^	42	3.82
88	Ramirez^88^	42	1.62
89	Durnig and Jungwirth^89^	42	2.80
90	Owsley and Zweifler^90^	42	2.21
91	Baker et al^91^	41	0.93
92	Lei et al^92^	41	2.56
93	De Cordier^93^	41	2.16
94	Hoefflin^94^	41	1.78
95	Abboushi et al^95^	40	4.44
96	Reilly et al^96^	38	6.33
97	Yoho et al^97^	38	2.38
98	Webb et al^98^	38	0.68
99	Jacono and Parikh^99^	38	3.80
100	Gupta et al^100^	37	7.40

References are provided in the Appendix, available online at www.aestheticsurgeryjournal.com.

The highest cited paper, authored by Stuzin et al, reviewed the relationship between the superficial and deep facial fascias and the relevance to rhytidectomy and aging.^10^ The most prolific author was Hamra with 10 first-author papers and 1 co-author paper featuring on the list (Appendix). This was followed by Baker with 5 first-author papers and 3 co-author papers, then Owsley with 5 first-author papers and 1 co-author paper (Appendix).

**Figure 2.**
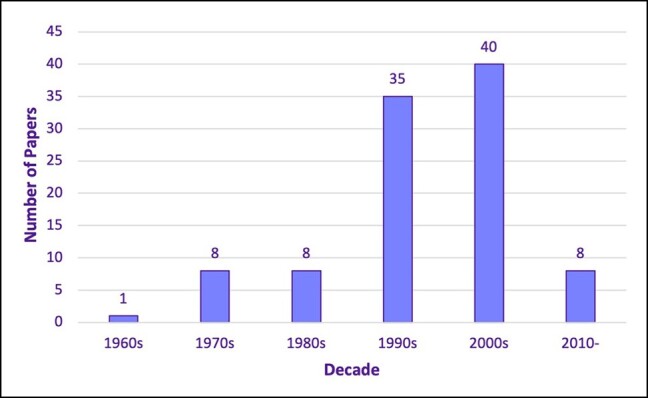
The 100 most cited papers—decade analysis.

Single-center study designs (*n* = 98) featured predominantly on the list compared with multicenter designs (*n* = 2). Both multicenter studies originated in the United States of America, which produced the highest output of papers overall (*n* = 75). This was followed by Australia (*n* = 5), then the United Kingdom (*n* = 4; see Table 2, which displays the countries contributing the 100 most cited papers). The decade between 2000 and 2010 yielded most of the highest cited papers on the list, accounting for 40 out of the top 100 articles (Figure 2). Eight papers formally acknowledged the receipt of funding. Ten papers explicitly stated receipt of no funding, and the remaining 82 papers did not declare funding status.

**Table 2.** Journals Contributing the 100 Most Cited Papers

Rank	Journal	No. of papers	Impact factor^a^
1	*Plastic and Reconstructive Surgery*	72	4.235
2	*Clinics in Plastic Surgery*	8	1.959
3	*Aesthetic Plastic Surgery*	4	1.798
4	*Aesthetic Surgery Journal*	3	
4	*British Journal of Plastic Surgery*	3	1.291
5	*Archives of Facial Plastic Surgery*	2	
5	*Dermatologic Surgery*	2	2.567
6	*Annals of Plastic Surgery*	1	1.354
6	*Cells Tissues Organs*	1	
6	*JAMA Facial Plastic Surgery*	1	
6	*Journal of Reconstructive Microsurgery*	1	
6	*Ophthalmic Plastic and Reconstructive Surgery*	1	
6	*Psychosomatic Medicine*	1	

^a^Impact factors as stated in the 2019 Journal Citation Reports (Clarivate Analytics, USA).

### Prevalent Research Themes

The top 100 articles originated from 13 journals. *Plastic and Reconstructive Surgery* (PRS) contributed the most papers (*n* = 72 papers), with the remaining journals contributing <10 papers each (see Table 3, which displays the journals contributing the 100 most cited papers).

**Table 3.** Countries Contributing the 100 Most Cited Papers

Rank	Country	No. of papers
1	USA	75
2	Australia	5
3	UK	4
4	Austria	2
4	Canada	2
4	France	2
4	Germany	2
4	Turkey	2
5	Belgium	1
5	Brazil	1
5	China	1
5	Israel	1
5	Singapore	1
5	Spain	1

**Figure 3.**
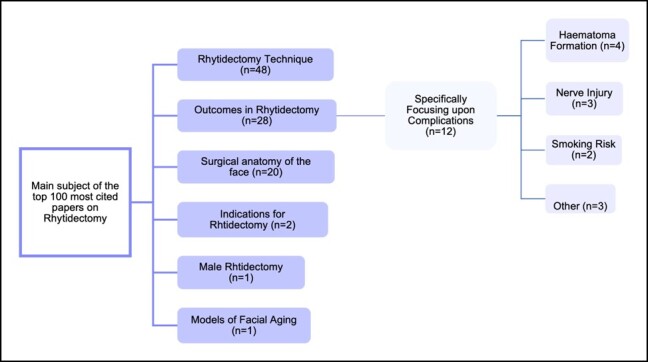
Subcategorization of papers—the main subject within the 100 most cited papers.

Rhytidectomy technique was the main subject in a significant proportion of papers (*n* = 48; Figure 3). Other prevalent research themes included rhytidectomy outcomes (*n* = 28) and facial anatomy (*n* = 20). One paper predominantly focused on male rhytidectomy (*n* = 1).^11^

### Methodological Quality

**Figure 4.**
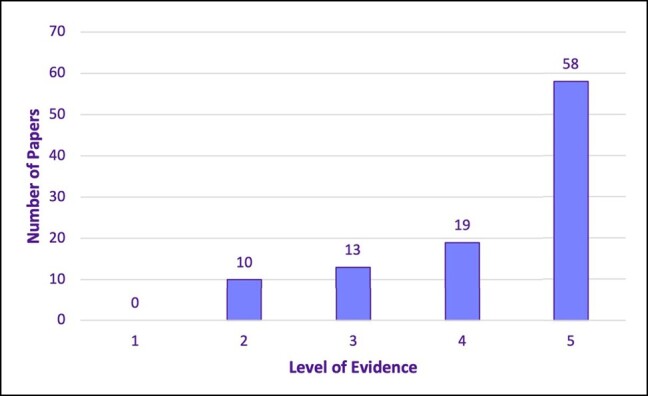
The 100 most cited papers—levels of evidence.

**Figure 5.**
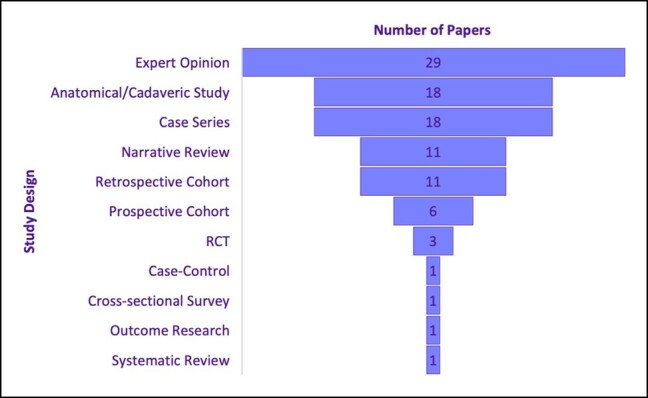
The 100 most cited papers—study designs.

Over half of the papers on the list were assessed to be OCEBM Level of Evidence 5 (*n* = 58), representative of the large majority of expert opinion papers (*n* = 29), anatomic/cadaveric (basic science) studies (*n* = 18), and narrative reviews (*n* = 11). Nineteen papers achieved Level of Evidence 4, while 13 achieved Level of Evidence 3, and 10 achieved Level of Evidence 2. No papers on the list attained Level of Evidence 1 (Figure 4). No firm level of evidence trends were observed with decade analysis. Study designs of the 100 most cited research are presented in Figure 5.

Clinical outcomes were reported in 84 of the top 100 most highly cited articles. Outcome measures were categorized in 83 studies. Subjective outcome measures were considered in 81 articles, including 56 papers comparing preoperative and postoperative photographic images. Ten papers incorporated PROMs (7 reporting quantitative outcomes and 3 reporting qualitative measures). No papers reported validated subjective or objective cosmetic outcome measures.

## DISCUSSION

To the best of our knowledge, this bibliometric analysis is the first of its kind to review and classify the highest cited rhytidectomy literature according to OCEBM methodological quality. Although studies largely failed to achieve the highest levels of evidence, the widespread adoption of subjective outcomes highlights the necessity of eliciting postprocedural patient satisfaction in the context of rhytidectomy outcomes.

This bibliometric analysis builds upon previous work, which used a machine-learning approach to derive topic trends for rhytidectomy abstracts through K-means cluster mapping.^3^ Machine learning has increasingly been applied in bibliometric analyses due to its scalability, open-access nature, and ease of use.^12,13^ However, limitations include inaccurate classification and poor model performance due to overfitting—a common phenomenon associated with machine learning use wherein the model learns the training data too closely and therefore when presented with new data, does not work as well.^13^ Therefore, this bibliometric analysis used traditional systematic review methods and extracted data from full texts, rather than abstracts, to provide a more comprehensive, accurate outline of impactful rhytidectomy research.

Broadly, our findings are representative of general trends across academic plastic surgery, whereby highly cited articles were mostly single-center, US-based, and published in PRS.^6^ Importantly, PROMs were lacking and overall levels of evidence were low.^6^ This is exemplified by the highest cited article in this analysis, studying the relevance of facial soft-tissue anatomy to the age-related stigmata of facial aging and rhytidectomy. Through fresh cadaver and intraoperative dissections, Stuzin et al^10^ characterized the arrangement of facial soft-tissue architecture into a series of concentric layers and determined the relationship between the superficial and deep facial fascias. Building upon description of the superficial musculoaponeurotic system (SMAS) by Mitz and Peyronie,^14^ the authors meticulously outlined the relationship of the SMAS to other facial structures and carefully described SMAS mobilization technique to minimize the risk of facial nerve injury.^10^ They also reported on the soft-tissue ligamentous support system of the face and its attenuation with aging.^10^ Although the paper is considered the anatomic basis of the “modern” facelift, as a cadaver study, the level of evidence was low, and there were no direct clinical or subjective outcomes relevant to the rhytidectomy procedure. Nevertheless, facial soft-tissue anatomy featured prominently in many of the other highly cited papers. Arguably, repositioning of these structures has been the most impactful on visual outcome of the face following rhytidectomy.^15^

Increased understanding of anatomical soft-tissue alterations contributing to facial aging has allowed the development of techniques to combat the specific anatomy leading to an aged appearance of the face.^16^ Authored by Hamra in 1992, the second most highly cited article elaborately describes the composite rhytidectomy technique.^17^ Building upon the standard SMAS facelift, Hamra refines his own description of the deep-plane rhytidectomy technique to address formation of the malar crescent caused by progressive orbicularis oculi ptosis in the aging face. In this paper, pioneering repositioning and fixation of the orbicularis oculi muscles in conjunction with en bloc repositioning of the SMAS, cheek fat pads, and cervical platysma in their original relationship with the overlying skin as a musculocutaneous flap in the deep-plane rhytidectomy technique, forms the hallmark of the composite facelift procedure.^17^ Hamra's technical quest to achieve a more natural and youthful appearance than the standard SMAS facelift gained acceptance and eminence.

The third most highly cited paper, entitled “The deep-plane rhytidectomy” elegantly describes an approach to target redundant laxity of the nasolabial folds.^18^ Written by Hamra in 1990, the technique preceded description of his composite rhytidectomy, and instigated novel inclusion of the malar fat pads into the facelift procedure.^18^ Differing from the SMAS lift originally described by Skoog in 1974,^19^ the operative technique in deep-plane rhytidectomy has been widely regarded to produce superior outcomes in the improvement of prominent nasolabial folds.^18^ While this likely accounts for the high citation number achieved, the procedure conferred a slightly higher risk of facial nerve injury,^18^ which may also partly explain the total number of citations.

The top 100 articles broadly capture the evolution of surgical rejuvenation techniques in the rhytidectomy procedure, from skin tension only techniques focusing on a variety of dissections and fixation planes (subcutaneous, sub-SMAS, subperiosteal), to commonplace repositioning or filling of deeper tissues prior to skin tightening and resection, and greater endeavors in combatting frequent sequelae of the “traditional facelift” such as optimization of tissue elevation vectors in a more anatomical vertical direction targeting “lateral sweep” and consideration of arcus marginalis release and orbital fat preservation (septal reset) in periorbital rejuvenation minimizing “hollow eyes.”^20^

Illustrated through several prominent highly cited articles featuring on the top 100 list, the quest for a more durable and less invasive means of facial rejuvenation has driven pursuit of procedures considered as alternatives to rhytidectomy and led to the development of ancillary concepts. One such paper by Ruiz-Esparza and Gomez,^21^ entitled “The medical facelift,” endeavored to evaluate the function of novel tissue tightening technology on facial skin laxity through the use of nonablative radiofrequency. Presenting a case series, the authors reported that nonablative radiofrequency technology is a safe and effective method to achieve tissue tightening of the face to correct excessive sagging, with the benefit of shorter downtime compared with traditional surgical rhytidectomy. Although ablative and nonablative resurfacing principles may result in improvements to the skin surface, adequate lifting of underlying ptotic tissues, crucial in achieving a youthful facial appearance, is not attained.^22^ Accruing 85 citations, this paper is likely representative of the peaked interest in minimally invasive approaches to facial rejuvenation.

Several further types of nonsurgical interventions and ancillary concepts have been described including injections with a variety of gels or autologous fat transfer through lipofilling.^22-24^ Although experts possessing an artistic touch may report pleasing results, the concepts in these procedures may result in unnatural contours with increased facial volume and visible center of gravity shifting to the lower third of the face.^22^ Such papers provided low contributions to the top 100 list, perhaps as such techniques are featured on newer emerging papers which generally are not cited until 1 to 2 years postpublications, with peak citation count at 3 to 10 years postpublication^25^ and therefore may not have yet accrued enough citations for inclusion within this bibliometric analysis.

The passage of sutures under the skin of the face and neck, utilizing threads in facelift procedures to compensate for redundant tissue sagging and flaccidity is not a new idea and can avoid large incisions and reduce recovery time.^26,27^ Together with traditional rhytidectomy incisions, suspension techniques are generally used to achieve better results and can be performed with autologous tissue, such as tendons or fascia, or prosthetic materials, including sutures, slings, or mesh.^26,28^ Among the many suspension techniques, subcutaneous suspension using SMAS as the fixation basis, with posterolateral vector tissue elevation, and subperiosteal detachment followed by en-bloc repositioning of structures using a purely vertical vector are concepts most widely developed.^29^ Suspension techniques have also been utilized to reposition and anchor the facial soft tissues to the temporal fascia or the periosteum in minimally invasive endoscopic procedures of the middle and lower facial thirds.^30^ Yet, there is a paucity of information in existing literature regarding efficacy, safety, durability, and possible complications of current clinical practices.^26^

In any elective cosmetic procedure, a serious complication, even when rare, is deemed unacceptable.^31,32^ Therefore, reducing operative risks in rhytidectomy by adopting methods for safe practice is a necessary prerequisite.^31,32^ Only 12 out of the 100 most cited papers focused on such specific complications and their prevention. Postrhytidectomy, hematoma remains the most common complication, often occurring within the first 24 h after the procedure.^31-34^ Hematoma formation has been significantly associated with neck undermining, systolic blood pressure, male gender, nonsteroidal anti-inflammatory or aspirin intake, and smoking. Hematoma may lead to prolonged facial swelling, skin necrosis, and even the potential for airway compromise.^31-34^ Adjuncts such as compressive dressings, drains, and fibrin glue have been tried to reduce the incidence of postrhytidectomy hematoma, though results have been mixed and often not statistically significant.^34,35^ However, the exclusion of adrenaline infiltration has been shown to significantly reduce hematoma formation requiring surgical evacuation, without change to the incidence of any other facelift complications.^34,35^ Though the paper by Grover et al showed the effectiveness of omitting vasoconstrictors in reducing hematoma, it has not been universally accepted as most surgeons prefer the “dry” field afforded by vasoconstrictors and hypotension.^34^

Although predominantly a procedure sought out and undertaken by females, more men are electing to undergo rhytidectomy than ever previously.^11^ Compared with the rhytidectomy procedure in females, the male rhytidectomy technique must follow a different approach throughout the surgical course for the preservation of hair follicles, restoration of a masculinized youthful appearance, and reduction of hematoma risk.^36^ This is comprehensively outlined by a single paper on the most cited list that predominantly focused on male rhytidectomy.^11^ The authors reported an increased incidence of large hematomas in male patients compared with females^11^; a finding that has since been confirmed.^26^ Interestingly, stringent perioperative blood pressure control has demonstrated reduced hematoma incidence in male patients,^37^ yet, not to a level comparable to or lesser than postrhytidectomy hematoma formation incidence in females.^37^ Further work is required to examine male-specific risks and complications, in addition to the incremental characterization and refinement of the male rhytidectomy surgical procedure.

Currently, a multitude of techniques are utilized for performing facelifts, yet there is no clear consensus as to which, if any, of these techniques may be most effective.^38^ Comparing facelifting techniques between lateral and standard SMAS facelifts with extended SMAS and composite rhytidectomies, Ivy et al^39^ featured on the top 100 list and presented a prospective study randomizing separate halves of 21 patients’ faces, 19 undergoing primary rhytidectomy. Although patients were followed up for 1-year postprocedure, with postoperative photographs taken at 6 and 12 months, the descriptive nature of outcomes presented, subjective comparison of postoperative photographs, and lack of PROMs render facilitation of comparison and evaluation between techniques challenging and complex. Interestingly, a retrospective cohort study also included in the list, undertaken by Kamer and Frankel^40^ comparing the tuck rate between SMAS rhytidectomy vs deep-plane rhytidectomy found that a tuck was required 71% less frequently following a deep-plane facelift than after a SMAS lift, although all rhytidectomies in the study were performed by 1 single surgeon and the authors acknowledged that a tuck procedure cannot reliably imply a less than optimal facelift in all cases. Recognizing variability among surgeons as an impeding factor in the comparison of facelifting techniques, the authors also highlighted the difficulty posed by factors such as the highly subjective nature of aesthetics, differences in patient anatomy, and specific patient desires.^40^

Although 10 papers in this bibliometric analysis reported PROMs, not surprisingly, none incorporated validated patient-reported outcomes. FACE-Q, the first validated PROM designed to measure a range of important outcomes for facial aesthetic surgery patients and developed for use in rhytidectomy alongside other facial aesthetic procedures was published in 2013.^41^ Since its inception to date, only 2 of the top 100 most highly cited papers accrued sufficient citations to be included on this list. Perhaps, in part, the lack of PROMs may be attributable to newer papers with lesser citations.^25^ Without reference to validated patient satisfaction indicators, it has been argued that clinical outcome measures are inadequate.^41^ Evaluation of PROMs is an urgent area requiring improvement in the rhytidectomy literature^41^ and clinical practice.^42^

Crucial for facilitating informed consent, shared decision making, patient choice, and benchmarking quality improvement in services, PROMs can help identify where surgical intervention in patients may be less likely to be of benefit,^41^ particularly important in rhytidectomy and other cosmetic procedures where appropriate patient selection is paramount.^42^ The collection of clinically meaningful data regarding aesthetic procedures through utilization of specific, valid, and relevant PROMs has been recommended by the American Society of Plastic Surgeons.^43^ Similarly, the routine reporting of PROMs preoperatively and postoperatively in facial aesthetic procedures including rhytidectomy has been advised by the Royal College of Surgeons in the United Kingdom^44^ and perhaps these proposals should be mandated in journals publishing research related to rhytidectomy.

Bibliometric analyses are subject to various potential inherent biases that have previously been well summarized^45^ and form possible limitations to this study. In-house review and author self-citation can result in an unconscious bias in addition to English language and national biases. Bandwagon bias, powerful person bias, and bias by omission are examples of conscious biases that can occur in attempts to attain a competitive publication edge. Therefore, automatically assuming that highly cited articles possess greater significance can result in incorrect conclusions. Instead, individual assessment of each article is recommended to critically appraise robustness of study methodology and formation of paper conclusions. Additionally, as cornerstone articles in rhytidectomy become accepted as assumed knowledge and therefore accrue lesser citations, they can become subject to obliteration by inclusion,^46^ which may provide an explanation for the absence of certain papers from this analysis.

Citation frequency analysis should be carefully interpreted when evaluating individual papers and study authors, with caution given to the aforementioned limitations.^25^ Nonetheless, citation frequency analysis has been recognized as a valid measure of evaluating research that is relied upon and assimilated by the scientific community within the specified field. The top 100 articles presented in this bibliometric analysis are considered immensely influential to the advance of modern-day practice in rhytidectomy, although other relevant papers that accrued lesser citations are likely to have been omitted. Nevertheless, this list, yielded by a comprehensive literature search, provides an excellent introduction to the classical rhytidectomy works. This bibliometric analysis provides a broad overview and historical perspective on pertinent topics gaining prominence, as well as highlighting deficiencies in studies’ methodological quality.

Despite market demands, social media trends, and ancillary procedures, the individual surgeon's preferred facelift technique has largely remained unchanged. We can speculate whether this reflects the surgeon's comfort level and satisfaction with a particular technique or is it, as we have presented here, that there is a lack of compelling evidence of 1 procedure over another.^38^ Although most surgeons have a “favorite” technique, collectively, the surgical community is limited by a lack of definitive data, facilitating the objective comparison of individual techniques.^47^ The various identical twins’ studies reported by Alpert et al,^48^ Antell et al,^49^ and Antell and Orseck^50^ further add that we lack compelling evidence that 1 technique is better than another. Increasing interest in ancillary concepts, such as lipofilling and other nonsurgical facial rejuvenation techniques, combined with the rhytidectomy procedure or as stand-alone alternatives to surgical facelifting, adds a further dimension and increases the complexity of technical and outcome-based comparison of facial rejuvenation. Higher quality evidence evaluating clinical and patient-reported outcomes through robust tools, such as FACE Q, is required to bring in higher level studies into the rhytidectomy literature forming a solid basis for evaluation of the various techniques.

## CONCLUSIONS

This extensive bibliometric analysis comprehensively examines the top 100 most highly cited papers in rhytidectomy and shows the evolution in the field over the past 6 decades. A multitude of refinements, safety considerations, and developments in rhytidectomy techniques are highlighted. Improvements in the quality of rhytidectomy literature must be sought by active prioritization of the publication of methodologically robust studies with higher OCEBM levels of evidence, such as well-designed randomized controlled trials or observational analytic studies. Furthermore, the adoption of validated PROMs designed for rhytidectomy is centrally important and crucial for aligning patient satisfaction with clinical outcomes and providing high-quality evidence-based patient care.

## Supplementary Material

ojad099_Supplementary_Data

## References

[1] Aesthetic Plastic Surgery National Databank Statistics 2020. Aesthet Surg J. 2021;41(Supplement_2):1–16. doi: 10.1093/asj/sjab178 33880491

[2] Sanan A , Most SP. Rhytidectomy (face-lift surgery). JAMA. 2018;320(22):2387. doi: 10.1001/jama.2018.17292 30535220

[3] Honeybrook A , Crowson M, Woodard C, Asaria J, Barrett D. The evolution and future of rhytidectomy literature: a bibliographic study. Am J Cosmetic Surg. 2018;36(2):85–90. doi: 10.1177/0748806818795555

[4] Moodley J , Singh V, Kagina BM, Abdullahi L, Hussey GD. A bibliometric analysis of cancer research in South Africa: study protocol. BMJ Open. 2015;5(2):e006913. doi: 10.1136/bmjopen-2014-006913 PMC4330326 25678542

[5] Sinha Y , Iqbal FM, Spence JN, Richard B. A bibliometric analysis of the 100 most-cited articles in rhinoplasty. Plast Reconstr Surg Glob Open. 2016;4(7):e820. doi: 10.1097/GOX.0000000000000834 27536499 PMC4977148

[6] Loonen MPJ , Hage JJ, Kon M. Plastic Surgery Classics: characteristics of 50 top-cited articles in four Plastic Surgery Journals since 1946. Plast Reconstr Surg. 2008;121(5):320e–327e. doi: 10.1097/PRS.0b013e31816b13a9 18453945

[7] Rifkin WJ , Yang JH, DeMitchell-Rodriguez E, Kantar RS, Diaz-Siso JR, Rodriguez ED. Levels of evidence in plastic surgery research: a 10-year bibliometric analysis of 18,889 publications from 4 major journals. Aesthet Surg J. 2020;40(2):220–227. doi: 10.1093/asj/sjz156 31119282

[8] Tahiri Y , Fleming TM, Greathouse T, Tholpady SS. Analysis of the 50 most cited papers in craniofacial surgery. J Craniomaxillofac Surg. 2015;43(10):2152–2157. doi: 10.1016/j.jcms.2015.09.011 26541748

[9] Centre for Evidence-Based Medicine . Oxford Centre for Evidence-Based Medicine: Levels of Evidence (March 2009). Centre for Evidence-Based Medicine (CEBM), University of Oxford. Accessed January 2, 2022. https://www.cebm.ox.ac.uk/resources/levels-of-evidence/oxford-centre-for-evidence-based-medicine-levels-of-evidence-march-2009

[10] Stuzin JM , Baker TJ, Gordon HL. The relationship of the superficial and deep facial fascias: relevance to rhytidectomy and aging. Plast Reconstr Surg. 1992;89(3):441–449. doi: 10.1097/00006534-199203000-00007 1741467

[11] Baker DC , Aston SJ, Guy CL, Rees TD. The male rhytidectomy. Plast Reconstr Surg. 1977;60(4):514–522. doi: 10.1097/00006534-197710000-00 909960

[12] Gupta D , Rinkle R. A study of big data evolution and research challenges. J Inform Sci. 2019;45(3):322–340. doi: 10.1177/0165551518789880

[13] Ngiam KY , Khor IW. Big data and machine learning algorithms for health-care delivery. Lancet Oncol. 2019;20(5):e262–e273. doi: 10.1016/S1470-2045(19)30149-4 31044724

[14] Mitz V , Peyronie M. The superficial musculo-aponeurotic system (SMAS) in the parotid and cheek area. Plast Reconstr Surg. 1976;58(1):80–88. doi: 10.1097/00006534-197607000-00013 935283

[15] Sykes JM , Riedler KL, Cotofana S, Palhazi P. Superficial and deep facial anatomy and its implications for rhytidectomy. Facial Plast Surg Clin North Am. 2020;28(3):243–251. doi: 10.1016/j.fsc.2020.03.005 32503712

[16] Swift A , Liew S, Weinkle S, Garcia JK, Silberberg MB. The facial aging process from the “inside out”. Aesthet Surg J. 2021;41(10):1107–1119. doi: 10.1093/asj/sjaa339 33325497 PMC8438644

[17] Hamra ST . Composite rhytidectomy. Plast Reconstr Surg. 1992;90(1):1–13. doi: 10.1097/00006534-199207000-00001 1615067

[18] Hamra ST . The deep-plane rhytidectomy. Plast Reconstr Surg. 1990;86(1):53–61. doi: 10.1097/00006534-199007000-00008 2359803

[19] Skoog T . Plastic Surgery: New Methods and Refinements. Saunders; 1974.

[20] Hamra ST . Frequent face lift sequelae: hollow eyes and the lateral sweep: cause and repair. Plast Reconstr Surg. 1998;102(5):1658–1666. doi: 10.1097/00006534-199810000-00052 9774028

[21] Ruiz-Esparza J , Gomez JB. The medical face lift: a noninvasive, nonsurgical approach to tissue tightening in facial skin using nonablative radiofrequency. Dermatol Surg. 2003;29(4):325–332; discussion 332. doi: 10.1046/j.1524-4725.2003.29080.x 12656808

[22] Sulamanidze MA , Paikidze TG, Sulamanidze GM, Neigel JM. Facial lifting with “APTOS” threads: featherlift. Otolaryngol Clin North Am. 2005;38(5):1109–1117. doi: 10.1016/j.otc.2005.05.005 16214576

[23] Paul MD . Barbed sutures for aesthetic facial plastic surgery: indications and techniques. Clin Plast Surg. 2008;35(3):451–461. doi: 10.1016/j.cps.2008.03.005 18558239

[24] Sulamanidze MA , Salti G, Mascetti M, Sulamanidze GM. Wire scalpel for surgical correction of soft tissue contour defects by subcutaneous dissection. Dermatol Surg. 2000;26(2):146–150; discussion 150-1. doi: 10.1046/j.1524-4725.2000.99127.x 10691945

[25] Baltussen A , Kindler CH. Citation classics in anesthetic journals. Anesth Analg. 2004;98(2):443–451. doi: 10.1213/01.ANE.0000096185.13474.0A 14742385

[26] Villa MT , White LE, Alam M, Yoo SS, Walton RL. Barbed sutures: a review of the literature. Plast Reconstr Surg. 2008;121(3):102–108. doi: 10.1097/01.prs.0000299452.24743.65 18317092

[27] Salasche SJ , Jarchow R, Feldman BD, Devine-Rust MJ, Adnot J. The suspension suture. J Dermatol Surg Oncol. 1987;13(9):973–978. doi: 10.1111/j.1524-4725.1987.tb00573.x 3305647

[28] Kaminer MS , Bogart M, Choi C, Wee SA. Long-term efficacy of anchored barbed sutures in the face and neck. Dermatol Surg. 2008;34(8):1041–1047. doi: 10.1111/j.1524-4725.2008.34203.x 18462425

[29] Berry MG , Davies D. Platysma-SMAS plication facelift. J Plast Reconstr Aesthet Surg. 2010;63(5):793–800. doi: 10.1016/j.bjps.2009.02.067 19328757

[30] Verpaele A , Tonnard P. Lower third of the face: indications and limitations of the minimal access cranial suspension lift. Clin Plast Surg. 2008;35(4):645–659. doi: 10.1016/j.cps.2008.04.001 18922317

[31] Baker SR , Moyer JS. Complications of rhytidectomy. Facial Plast Surg Clin North Am. 2005;13(3):469–478. doi: 10.1016/j.fsc.2005.04.005 16085292

[32] Gupta V , Winocour J, Shi H, Shack RB, Grotting JC, Higdon KK. Preoperative risk factors and complication rates in facelift: analysis of 11,300 patients. Aesthet Surg J. 2016;36(1):1–13. doi: 10.1093/asj/sjv162 26578747

[33] Grover R , Jones BM, Waterhouse N. The prevention of haematoma following rhytidectomy: a review of 1078 consecutive facelifts. Br J Plast Surg. 2001;54(6):481–486. doi: 10.1054/bjps.2001.3623 11513508

[34] Niamtu J 3rd . Expanding hematoma in face-lift surgery: literature review, case presentations, and caveats. Dermatol Surg. 2005;31(9 Pt 1):1134–1144; discussion 1144. doi: 10.1097/00042728-200509000-00012 16164866

[35] Jones BM , Grover R. Avoiding hematoma in cervicofacial rhytidectomy: a personal 8-year quest. Reviewing 910 patients. Plast Reconstr Surg. 2004;113(1):381–387; discussion 388-390. doi: 10.1097/01.PRS.0000097291.15196.78 14707663

[36] Rohrich RJ , Stuzin JM, Ramanadham S, Costa C, Dauwe PB. The modern male rhytidectomy: lessons learned. Plast Reconstr Surg. 2017;139(2):295–307. doi: 10.1097/PRS.0000000000003008 28125532

[37] Baker DC , Stefani WA, Chiu ES. Reducing the incidence of hematoma requiring surgical evacuation following male rhytidectomy: a 30-year review of 985 cases. Plast Reconstr Surg. 2005;116(7):1973–1985; discussion 1986-1987. doi: 10.1097/01.prs.0000191182.70617.e9 16327611

[38] Sinclair NR , Kochuba A, Coombs DM, et al Face lift practice patterns: an American Society of Plastic Surgeons Member Survey, 2000 and 2020. How much have we changed? Plast Reconstr Surg. 2022;149(6):1096e–1105e. doi: 10.1097/PRS.0000000000009097 35383689

[39] Ivy EJ , Lorenc ZP, Aston SJ. Is there a difference? A prospective study comparing lateral and standard SMAS face lifts with extended SMAS and composite rhytidectomies. Plast Reconstr Surg. 1996;98(7):1135–1143; discussion 1144-1147. doi: 10.1097/00006534-199612000-00001 8942899

[40] Kamer FM , Frankel AS. SMAS rhytidectomy versus deep plane rhytidectomy: an objective comparison. Plast Reconstr Surg. 1998;102(3):878–881. doi: 10.1097/00006534-199809030-00041 9727459

[41] RCSEng, The Royal College of Surgeons of England . Use of patient reported outcome measures within clinical practice. 2020. https://www.rcseng.ac.uk/standards-and-research/standards-and-guidance/service-standards/cosmetic-surgery/clinical-quality-and-outcomes/patient-reported-outcome-measures/

[42] Urso-Baiarda F , Townley W, Branford O, Rohrich R. PROMs – king of outcome assessment tools: understanding how patients feel about their cosmetic surgery. 2015. Accessed February 11, 2015. https://prsonallyspeaking.wordpress.com/2015/02/11/proms-king-of-outcome-assessment-tools-understanding-how-patients-feel-about-their-cosmetic-surgery/

[43] ASPS recommends the use of plastic surgery specific patient-reported outcome measures. @ASPS_News. 2020. Accessed November 8, 2023.

[44] The Royal College of Surgeons of England . Patient reported outcome measures 2020. Accessed November 8, 2023.

[45] Dumont JE . The bias of citations. Trends Biochem Sci. 1989;14(8):327–328. doi: 10.1016/0968-0004(89)90164-3 2799904

[46] Garfield E . 100 citation classics from the Journal of the American Medical Association. JAMA. 1987;257(1):52–59. doi: 10.1001/jama.1987.03390010056028 3537352

[47] Nahai F . Complications in aesthetic surgery: evaluating the data. Aesthet Surg J. 2019;39(9):1035–1036. doi: 10.1093/asj/sjz092. 31329821

[48] Alpert BS , Baker DC, Hamra ST, Owsley JQ, Ramirez O. Identical twin face lifts with differing techniques: a 10-year follow-up. Plast Reconstr Surg. 2009;123(3):1025–1033. doi: 10.1097/PRS.0b013e31819ba755 19319071

[49] Antell DE , May JM, Bonnano MJ, Lee NY. A comparison of the full and short-scar face-lift incision techniques in multiple sets of identical twins. Plast Reconstr Surg. 2016;137(6):1707–1714. doi: 10.1097/PRS.0000000000002229 26890507

[50] Antell DE , Orseck MJ. A comparison of face lift techniques in eight consecutive sets of identical twins. Plast Reconstr Surg. 2007;120(6):1667–1673. doi: 10.1097/01.prs.0000288016.24950.20 18040204

