# Supplementary material for: A Bibliometric Analysis of the Highest Cited Rhytidectomy Literature
Source: Aesthet Surg J Open Forum. 2023 Oct 31;5:ojad099. doi: 10.1093/asjof/ojad099 (PMC10702622; doi:10.1093/asjof/ojad099)
Supplement: ojad099_Supplementary_Data [file ojad099_supplementary_data.docx]

**Appendix.** Complete Citations Are Provided for All of the 100 Most-Cited Papers

1. Stuzin JM, Baker TJ, Gordon HL. The relationship of the superficial and deep facial fascias: relevance to rhytidectomy and aging. *Plast Reconstr Surg*. 1992;89(3):441-451.
2. Hamra ST. Composite rhytidectomy. *Plast Reconstr Surg*. 1992;90(1):1-13. doi:10.1097/00006534-199207000-00001
3. Hamra ST. The deep-plane rhytidectomy. *Plast Reconstr Surg*. 1990;86(1):53-63.
4. Hamra ST. Arcus marginalis release and orbital fat preservation in midface rejuvenation. *Plast Reconstr Surg*. 1995;96(2):354-362. doi:10.1097/00006534-199508000-00014
5. Mendelson BC, Muzaffar AR, Adams WP Jr. Surgical anatomy of the midcheek and malar mounds. *Plast Reconstr Surg*. 2002;110(3):885-911. doi:10.1097/00006534-200209010-00026
6. Baker DC, Conley J. Avoiding facial nerve injuries in rhytidectomy. Anatomical variations and pitfalls. *Plast Reconstr Surg*. 1979;64(6):781-795. doi:10.1097/00006534-197912000-00005
7. Alsarraf R. Outcomes Research in Facial Plastic Surgery: A Review and New Directions. *Aesthetic Plast Surg*. 2020;44(4):1210-1215. doi:10.1007/s00266-020-01809-9
8. Owsley JQ. Lifting the malar fat pad for correction of prominent nasolabial folds. *Plast Reconstr Surg*. 1993;91(3):463-476.
9. Moss CJ, Mendelson BC, Taylor GI. Surgical anatomy of the ligamentous attachments in the temple and periorbital regions. *Plast Reconstr Surg*. 2000;105(4):1475-1498.
10. Hester TR Jr, Codner MA, McCord CD, Nahai F, Giannopoulos A. Evolution of technique of the direct transblepharoplasty approach for the correction of lower lid and midfacial aging: maximizing results and minimizing complications in a 5-year experience. *Plast Reconstr Surg*. 2000;105(1):393-408. doi:10.1097/00006534-200001000-00063
11. Hamra ST. The zygorbicular dissection in composite rhytidectomy: an ideal midface plane. *Plast Reconstr Surg*. 1998;102(5):1646-1657. doi:10.1097/00006534-199810000-00051
12. Tonnard P, Verpaele A, Monstrey S, et al. Minimal access cranial suspension lift: a modified S-lift. *Plast Reconstr Surg*. 2002;109(6):2074-2086. doi:10.1097/00006534-200205000-00046
13. Kosowski TR, McCarthy C, Reavey PL, et al. A systematic review of patient-reported outcome measures after facial cosmetic surgery and/or nonsurgical facial rejuvenation. *Plast Reconstr Surg*. 2009;123(6):1819-1827. doi:10.1097/PRS.0b013e3181a3f361
14. Hamra ST. The role of orbital fat preservation in facial aesthetic surgery. A new concept. *Clin Plast Surg*. 1996;23(1):17-28.
15. Stuzin JM, Baker TJ, Gordon HL, Baker TM. Extended SMAS dissection as an approach to midface rejuvenation. *Clin Plast Surg*. 1995;22(2):295-311.
16. Gosain AK, Klein MH, Sudhakar PV, Prost RW. A volumetric analysis of soft-tissue changes in the aging midface using high-resolution MRI: implications for facial rejuvenation. *Plast Reconstr Surg*. 2005;115(4):1143-1155. doi:10.1097/01.prs.0000156333.57852.2f
17. Grover R, Jones BM, Waterhouse N. The prevention of haematoma following rhytidectomy: a review of 1078 consecutive facelifts. *Br J Plast Surg*. 2001;54(6):481-486. doi:10.1054/bjps.2001.3623
18. Chang LD, Buncke G, Slezak S, Buncke HJ. Cigarette smoking, plastic surgery, and microsurgery. *J Reconstr Microsurg*. 1996;12(7):467-474. doi:10.1055/s-2007-1006620
19. Ramirez OM. Endoscopic full facelift. *Aesthetic Plast Surg*. 1994;18(4):363-371. doi:10.1007/BF00451341
20. Connell BF. Contouring the neck in rhytidectomy by lipectomy and a muscle sling. *Plast Reconstr Surg*. 1978;61(3):376-383. doi:10.1097/00006534-197803000-00011
21. Riefkohl R, Wolfe JA, Cox EB, McCarty KS Jr. Association between cutaneous occlusive vascular disease, cigarette smoking, and skin slough after rhytidectomy. *Plast Reconstr Surg*. 1986;77(4):592-595. doi:10.1097/00006534-198604000-00013
22. Zhang HM, Yan YP, Qi KM, Wang JQ, Liu ZF. Anatomical structure of the buccal fat pad and its clinical adaptations. *Plast Reconstr Surg*. 2002;109(7):2509-2520. doi:10.1097/00006534-200206000-00052
23. Marchac D, Sándor G. Face lifts and sprayed fibrin glue: an outcome analysis of 200 patients. *Br J Plast Surg*. 1994;47(5):306-309. doi:10.1016/0007-1226(94)90087-6
24. Kikkawa DO, Lemke BN, Dortzbach RK. Relations of the superficial musculoaponeurotic system to the orbit and characterization of the orbitomalar ligament. *Ophthalmic Plast Reconstr Surg*. 1996;12(2):77-88. doi:10.1097/00002341-199606000-00001
25. Barton FE Jr. Rhytidectomy and the nasolabial fold. *Plast Reconstr Surg*. 1992;90(4):601-607. doi:10.1097/00006534-199210000-00008
26. Ghassemi A, Prescher A, Riediger D, Axer H. Anatomy of the SMAS revisited. *Aesthetic Plast Surg*. 2003;27(4):258-264. doi:10.1007/s00266-003-3065-3
27. Ruiz-Esparza J, Gomez JB. The medical face lift: a noninvasive, nonsurgical approach to tissue tightening in facial skin using nonablative radiofrequency. *Dermatol Surg*. 2003;29(4):325-332. doi:10.1046/j.1524-4725.2003.29080.x
28. Hamra ST. Repositioning the orbicularis oculi muscle in the composite rhytidectomy. *Plast Reconstr Surg*. 1992;90(1):14-22. doi:10.1097/00006534-199207000-00002
29. Ivy EJ, Lorenc ZP, Aston SJ. Is there a difference? A prospective study comparing lateral and standard SMAS face lifts with extended SMAS and composite rhytidectomies. *Plast Reconstr Surg*. 1996;98(7):1135-1147. doi:10.1097/00006534-199612000-00001
30. Barton FE Jr. The SMAS and the nasolabial fold. *Plast Reconstr Surg*. 1992;89(6):1054-1059.
31. Owsley JQ Jr. Platysma-fascial rhytidectomy: a preliminary report. *Plast Reconstr Surg*. 1977;60(6):843-850. doi:10.1097/00006534-197712000-00001
32. Little JW. Volumetric perceptions in midfacial aging with altered priorities for rejuvenation. *Plast Reconstr Surg*. 2000;105(1):252-289. doi:10.1097/00006534-200001000-00043
33. Jones BM, Grover R. Avoiding hematoma in cervicofacial rhytidectomy: a personal 8-year quest. Reviewing 910 patients. *Plast Reconstr Surg*. 2004;113(1):381-390. doi:10.1097/01.PRS.0000097291.15196.78
34. Gosain AK, Amarante MT, Hyde JS, Yousif NJ. A dynamic analysis of changes in the nasolabial fold using magnetic resonance imaging: implications for facial rejuvenation and facial animation surgery. *Plast Reconstr Surg*. 1996;98(4):622-636. doi:10.1097/00006534-199609001-00005
35. Ramirez OM. Three-dimensional endoscopic midface enhancement: a personal quest for the ideal cheek rejuvenation. *Plast Reconstr Surg*. 2002;109(1):329-349. doi:10.1097/00006534-200201000-00052
36. Mendelson BC, Freeman ME, Wu W, Huggins RJ. Surgical anatomy of the lower face: the premasseter space, the jowl, and the labiomandibular fold. *Aesthetic Plast Surg*. 2008;32(2):185-195. doi:10.1007/s00266-007-9060-3
37. Tellioğlu AT, Tekdemir I, Erdemli EA, Tüccar E, Ulusoy G. Temporoparietal fascia: an anatomic and histologic reinvestigation with new potential clinical applications. *Plast Reconstr Surg*. 2000;105(1):40-45. doi:10.1097/00006534-200001000-00007
38. Hamra ST. The role of the septal reset in creating a youthful eyelid-cheek complex in facial rejuvenation. *Plast Reconstr Surg*. 2004;113(7):2124-2144. doi:10.1097/01.prs.0000122410.19952.e7
39. Owsley JQ Jr. SMAS-platysma facelift. A bidirectional cervicofacial rhytidectomy. *Clin Plast Surg*. 1983;10(3):429-440.
40. Mendelson BC. Surgery of the superficial musculoaponeurotic system: principles of release, vectors, and fixation. *Plast Reconstr Surg*. 2001;107(6):1545-1561.
41. Lemmon ML, Hamra ST. Skoog rhytidectomy: a five-year experience with 577 patients. *Plast Reconstr Surg*. 1980;65(3):283-297.
42. McKinney P, Katrana DJ. Prevention of injury to the great auricular nerve during rhytidectomy. *Plast Reconstr Surg*. 1980;66(5):675-679. doi:10.1097/00006534-198011000-00001
43. Baker DC, Stefani WA, Chiu ES. Reducing the incidence of hematoma requiring surgical evacuation following male rhytidectomy: a 30-year review of 985 cases. *Plast Reconstr Surg*. 2005;116(7):1973-1987. doi:10.1097/01.prs.0000191182.70617.e9
44. Stuzin JM. Restoring facial shape in face lifting: the role of skeletal support in facial analysis and midface soft-tissue repositioning. *Plast Reconstr Surg*. 2007;119(1):362-376. doi:10.1097/01.prs.0000251092.82860.25
45. Labbé D, Franco RG, Nicolas J. Platysma suspension and platysmaplasty during neck lift: anatomical study and analysis of 30 cases. *Plast Reconstr Surg*. 2006;117(6):2001-2010. doi:10.1097/01.prs.0000218972.75144.9c
46. Byrd HS, Andochick SE. The deep temporal lift: a multiplanar, lateral brow, temporal, and upper face lift. *Plast Reconstr Surg*. 1996;97(5):928-937. doi:10.1097/00006534-199604001-00007
47. Rohrich RJ, Beran SJ. Evolving fixation methods in endoscopically assisted forehead rejuvenation: controversies and rationale. *Plast Reconstr Surg*. 1997;100(6):1575-1584. doi:10.1097/00006534-199711000-00032
48. Wong CH, Mendelson B. Facial soft-tissue spaces and retaining ligaments of the midcheek: defining the premaxillary space. *Plast Reconstr Surg*. 2013;132(1):49-56. doi:10.1097/PRS.0b013e3182910a57
49. Baker TJ, Gordon HL, Mosienko P. Rhytidectomy: a statistical analysis. *Plast Reconstr Surg*. 1977;59(1):24-30.
50. Trussler AP, Stephan P, Hatef D, Schaverien M, Meade R, Barton FE. The frontal branch of the facial nerve across the zygomatic arch: anatomical relevance of the high-SMAS technique. *Plast Reconstr Surg*. 2010;125(4):1221-1229. doi:10.1097/PRS.0b013e3181d18136
51. Daane SP, Owsley JQ. Incidence of cervical branch injury with "marginal mandibular nerve pseudo-paralysis" in patients undergoing face lift. *Plast Reconstr Surg*. 2003;111(7):2414-2418. doi:10.1097/01.PRS.0000061004.74788.33
52. Hamra ST. Frequent face lift sequelae: hollow eyes and the lateral sweep: cause and repair. *Plast Reconstr Surg*. 1998;102(5):1658-1666. doi:10.1097/00006534-199810000-00052
53. Rees TD, Barone CM, Valauri FA, Ginsberg GD, Nolan WB 3rd. Hematomas requiring surgical evacuation following face lift surgery. *Plast Reconstr Surg*. 1994;93(6):1185-1190. doi:10.1097/00006534-199405000-00012
54. Baker DC. Complications of cervicofacial rhytidectomy. *Clin Plast Surg*. 1983;10(3):543-562.
55. Marten TJ. High SMAS facelift: combined single flap lifting of the jawline, cheek, and midface. *Clin Plast Surg*. 2008;35(4):569-vii. doi:10.1016/j.cps.2008.04.002
56. Lambros V. Models of facial aging and implications for treatment. *Clin Plast Surg*. 2008;35(3):319-317. doi:10.1016/j.cps.2008.02.012
57. Ozdemir R, Kilinç H, Unlü RE, Uysal AC, Sensöz O, Baran CN. Anatomicohistologic study of the retaining ligaments of the face and use in face lift: retaining ligament correction and SMAS plication. *Plast Reconstr Surg*. 2002;110(4):1134-1149. doi:10.1097/01.PRS.0000021442.30272.0E
58. Seeley BM, Denton AB, Ahn MS, Maas CS. Effect of homeopathic Arnica montana on bruising in face-lifts: results of a randomized, double-blind, placebo-controlled clinical trial. *Arch Facial Plast Surg*. 2006;8(1):54-59. doi:10.1001/archfaci.8.1.54
59. Rohrich RJ, Ghavami A, Lemmon JA, Brown SA. The individualized component face lift: developing a systematic approach to facial rejuvenation. *Plast Reconstr Surg*. 2009;123(3):1050-1063. doi:10.1097/PRS.0b013e31819c91b0
60. De La Plaza R, Valiente E, Arroyo JM. Supraperiosteal lifting of the upper two-thirds of the face [published correction appears in Br J Plast Surg 1992 Feb-Mar;45(2):185] [published correction appears in Br J Plast Surg 1992 Jan;45(1):80]. *Br J Plast Surg*. 1991;44(5):325-332. doi:10.1016/0007-1226(91)90143-8
61. Goin MK, Burgoyne RW, Goin JM, Staples FR. A prospective psychological study of 50 female face-lift patients. *Plast Reconstr Surg*. 1980;65(4):436-442. doi:10.1097/00006534-198004000-00007
62. Har-Shai Y, Bodner SR, Egozy-Golan D, et al. Mechanical properties and microstructure of the superficial musculoaponeurotic system. *Plast Reconstr Surg*. 1996;98(1):59-73. doi:10.1097/00006534-199607000-00009
63. Gassner HG, Rafii A, Young A, Murakami C, Moe KS, Larrabee WF Jr. Surgical anatomy of the face: implications for modern face-lift techniques. *Arch Facial Plast Surg*. 2008;10(1):9-19. doi:10.1001/archfacial.2007.16
64. Kamer FM, Frankel AS. SMAS rhytidectomy versus deep plane rhytidectomy: an objective comparison. *Plast Reconstr Surg*. 1998;102(3):878-881. doi:10.1097/00006534-199809030-00041
65. Mendelson BC. Extended sub-SMAS dissection and cheek elevation. *Clin Plast Surg*. 1995;22(2):325-339.
66. Jones BM, Grover R. Reducing complications in cervicofacial rhytidectomy by tumescent infiltration: a comparative trial evaluating 678 consecutive face lifts. *Plast Reconstr Surg*. 2004;113(1):398-403. doi:10.1097/01.PRS.0000097297.89136.8D
67. Hamra ST. A study of the long-term effect of malar fat repositioning in face lift surgery: short-term success but long-term failure. *Plast Reconstr Surg*. 2002;110(3):940-959. doi:10.1097/00006534-200209010-00035
68. Sasaki GH, Cohen AT. Meloplication of the malar fat pads by percutaneous cable-suture technique for midface rejuvenation: outcome study (392 cases, 6 years' experience). *Plast Reconstr Surg*. 2002;110(2):635-657. doi:10.1097/00006534-200208000-00042
69. Owsley JQ, Fiala TG. Update: lifting the malar fat pad for correction of prominent nasolabial folds. *Plast Reconstr Surg*. 1997;100(3):715-722. doi:10.1097/00006534-199709000-00029
70. Guyuron B, Vaughan C. A comparison of absorbable and nonabsorbable suture materials for skin repair. *Plast Reconstr Surg*. 1992;89(2):234-236. doi:10.1097/00006534-199202000-00005
71. Berner RE, Morain WD, Noe JM. Postoperative hypertension as an etiological factor in hematoma after rhytidectomy. Prevention with chlorpromazine. *Plast Reconstr Surg*. 1976;57(3):314-319. doi:10.1097/00006534-197603000-00006
72. Rees TD, Lee YC, Coburn RJ. Expanding hematoma after rhytidectomy. A retrospective study. *Plast Reconstr Surg*. 1973;51(2):149-153. doi:10.1097/00006534-197302000-00007
73. Paul MD, Calvert JW, Evans GR. The evolution of the midface lift in aesthetic plastic surgery. *Plast Reconstr Surg*. 2006;117(6):1809-1827. doi:10.1097/01.prs.0000218839.55122.c0
74. Ramirez OM. The subperiosteal rhytidectomy: the third-generation face-lift. *Ann Plast Surg*. 1992;28(3):218-234. doi:10.1097/00000637-199203000-00005
75. Warren RJ, Aston SJ, Mendelson BC. Face lift. *Plast Reconstr Surg*. 2011;128(6):747e-764e. doi:10.1097/PRS.0b013e318230c939
76. Fezza JP, Cartwright M, Mack W, Flaharty P. The use of aerosolized fibrin glue in face-lift surgery. *Plast Reconstr Surg*. 2002;110(2):658-666. doi:10.1097/00006534-200208000-00044
77. Hamra ST. The deep-plane rhytidectomy. *Plast Reconstr Surg*. 1990;86(1):61-63.
78. Jones BM, Grover R, Hamilton S. The efficacy of surgical drainage in cervicofacial rhytidectomy: a prospective, randomized, controlled trial. *Plast Reconstr Surg*. 2007;120(1):263-270. doi:10.1097/01.prs.0000264395.38684.5a
79. Stuzin JM, Baker TJ, Baker TM. Refinements in face lifting: enhanced facial contour using vicryl mesh incorporated into SMAS fixation. *Plast Reconstr Surg*. 2000;105(1):290-301. doi:10.1097/00006534-200001000-00046
80. Gunter JP, Hackney FL. A simplified transblepharoplasty subperiosteal cheek lift. *Plast Reconstr Surg*. 1999;103(7):2029-2041. doi:10.1097/00006534-199906000-00036
81. Freiberg A, Giguère D, Ross DC, Taylor JR, Bell T, Kerluke LD. Are patients satisfied with results from residents performing aesthetic surgery?. *Plast Reconstr Surg*. 1997;100(7):1824-1833. doi:10.1097/00006534-199712000-00029
82. Pitanguy I. Indications for and treatment of frontal and glabellar wrinkles in an analysis of 3,404 consecutive cases of rhytidectomy. *Plast Reconstr Surg*. 1981;67(2):157-168.
83. Teimourian B. Face and neck suction-assisted lipectomy associated with rhytidectomy. *Plast Reconstr Surg*. 1983;72(5):627-633. doi:10.1097/00006534-198311000-00006
84. Conway H. The surgical face lift--rhytidectomy. *Plast Reconstr Surg*. 1970;45(2):124-130. doi:10.1097/00006534-197002000-00003
85. Macchi V, Tiengo C, Porzionato A, et al. Histotopographic study of the fibroadipose connective cheek system. *Cells Tissues Organs*. 2010;191(1):47-56. doi:10.1159/000226276
86. Barton FE Jr, Gyimesi IM. Anatomy of the nasolabial fold. *Plast Reconstr Surg*. 1997;100(5):1276-1280. doi:10.1097/00006534-199710000-00032
87. Ramirez OM. Endoscopic subperiosteal browlift and facelift. *Clin Plast Surg*. 1995;22(4):639-660.
88. Baker DC, Aston SJ, Guy CL, Rees TD. The male rhytidectomy. *Plast Reconstr Surg*. 1977;60(4):514-522. doi:10.1097/00006534-197710000-00003
89. Lei T, Xu DC, Gao JH, et al. Using the frontal branch of the superficial temporal artery as a landmark for locating the course of the temporal branch of the facial nerve during rhytidectomy: an anatomical study. *Plast Reconstr Surg*. 2005;116(2):623-630. doi:10.1097/01.prs.0000174001.95115.9e
90. De Cordier BC, de la Torre JI, Al-Hakeem MS, et al. Rejuvenation of the midface by elevating the malar fat pad: review of technique, cases, and complications. *Plast Reconstr Surg*. 2002;110(6):1526-1540. doi:10.1097/01.PRS.0000029816.67278.1B
91. Hoefflin SM. The extended supraplatysmal plane (ESP) face lift. *Plast Reconstr Surg*. 1998;101(2):494-503. doi:10.1097/00006534-199802000-00039
92. Abboushi N, Yezhelyev M, Symbas J, Nahai F. Facelift complications and the risk of venous thromboembolism: a single center's experience. *Aesthet Surg J*. 2012;32(4):413-420. doi:10.1177/1090820X12442213
93. Durnig P, Jungwirth W. Low-molecular-weight heparin and postoperative bleeding in rhytidectomy. *Plast Reconstr Surg*. 2006;118(2):502-509. doi:10.1097/01.prs.0000228180.78071.44
94. Owsley JQ Jr, Zweifler M. Midface lift of the malar fat pad: technical advances. *Plast Reconstr Surg*. 2002;110(2):674-687. doi:10.1097/00006534-200208000-00048
95. Matarasso A, Terino EO. Forehead-brow rhytidoplasty: reassessing the goals. *Plast Reconstr Surg*. 1994;93(7):1378-1391.
96. Reilly MJ, Tomsic JA, Fernandez SJ, Davison SP. Effect of facial rejuvenation surgery on perceived attractiveness, femininity, and personality. *JAMA Facial Plast Surg*. 2015;17(3):202-207. doi:10.1001/jamafacial.2015.0158
97. Yoho RA, Romaine JJ, O'Neil D. Review of the liposuction, abdominoplasty, and face-lift mortality and morbidity risk literature [published correction appears in Dermatol Surg. 2005 Sep;31(9 Pt 1):1158]. *Dermatol Surg*. 2005;31(7 Pt 1):733-743. doi:10.1097/00042728-200507000-00001
98. Webb WL Jr, Slaughter R, Meyer E, Edgerton M. Mechanisms Of Psychosocial Adjustment In Patients Seeking " Face-Lift" Operation. *Psychosom Med*. 1965;27:183-192. doi:10.1097/00006842-196503000-00011
99. Gupta V, Winocour J, Shi H, Shack RB, Grotting JC, Higdon KK. Preoperative Risk Factors and Complication Rates in Facelift: Analysis of 11,300 Patients. *Aesthet Surg J*. 2016;36(1):1-13. doi:10.1093/asj/sjv162
100. Jacono AA, Parikh SS. The minimal access deep plane extended vertical facelift. *Aesthet Surg J*. 2011;31(8):874-890. doi:10.1177/1090820X11424146
